# Illuminating the subcellular maze: fluorescence-activated organelle sorting in plant sciences

**DOI:** 10.1093/jxb/eraf490

**Published:** 2025-11-15

**Authors:** Vladimír Skalický, Ioanna Antoniadi, Karin Ljung, Ondřej Novák

**Affiliations:** Laboratory of Growth Regulators, Faculty of Science, Palacký University and Institute of Experimental Botany, Czech Academy of Sciences, Šlechtitelů 27, Olomouc CZ-77900, Czech Republic; Umeå Plant Science Centre, Department of Forest Genetics and Plant Physiology, Swedish University of Agricultural Sciences, Umeå SE-901 83, Sweden; Umeå Plant Science Centre, Department of Forest Genetics and Plant Physiology, Swedish University of Agricultural Sciences, Umeå SE-901 83, Sweden; Umeå Plant Science Centre, Department of Forest Genetics and Plant Physiology, Swedish University of Agricultural Sciences, Umeå SE-901 83, Sweden; Laboratory of Growth Regulators, Faculty of Science, Palacký University and Institute of Experimental Botany, Czech Academy of Sciences, Šlechtitelů 27, Olomouc CZ-77900, Czech Republic; Cardiff University, UK

**Keywords:** Fluorescence-activated organelle sorting (FAOS), plant flow cytometry, plant organelle analysis, subcellular fractionation

## Abstract

The isolation of organelles is critical for gaining a deeper understanding of their functions in intracellular processes, not only at the cellular but also at the multicellular, organ, and organism levels. Isolating them into pure fractions allows for the reduction of sample complexity, thereby ensuring high quality downstream analysis, such as in protein localization studies. Since the mid-20th century, new methods of subcellular fractionation have constantly emerged. Conventional fractionation approaches based on (ultra)centrifugation typically focus on isolating only one type of organelle. Moreover, their resolving power may be inadequate for improving the limit of detection of downstream applications. Fluorescence-activated organelle sorting (FAOS) is a versatile and advanced technique that is gaining popularity due to its high efficiency. This efficiency refers to the ability to monitor organelle isolation live and to sort multiple organelle populations simultaneously from a single sample. This review offers an overview of the usage of FAOS and highlights its promising prospects within the realm of plant sciences. FAOS shows great potential for applications in both the functional and structural analysis of plant organelles while serving as a valuable isolation tool for downstream applications, including ‘omics’ studies.

## Teaser

We present an overview of how fluorescence-activated sorting enhances plant organelle isolation, showcasing its advantages, recent achievements, and future applications in plant research.

## Introduction

Cells represent the smallest independent units of living systems. Nevertheless, the intracellular space is further subdivided into compartments or organelles, each serving distinct functions. Subcellular compartmentation in eukaryotic cells facilitates increased membrane surfaces, segregating opposing reactions and allowing for organelle specialization. Most organelles are surrounded by one or two phospholipid bilayers, enabling the specific creation and maintenance of microenvironments, such as pH, ion presence, or substrate availability. However, membrane-less organelles and structures also occur in plant cells, such as the nucleolus, ribosomes, and oil or protein bodies. Organelle functions depend on their specific protein/enzyme, genetic, and/or lipid compositions (Lee *et al.*, 2010).

**Fig. 1.**
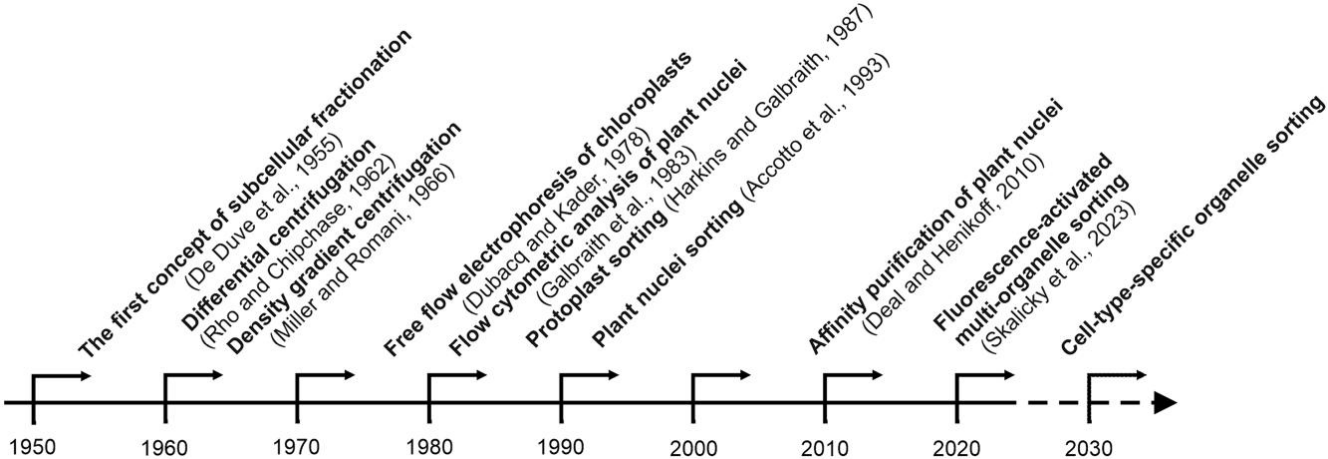
Evolution of organelle isolation techniques in plant science: a decades-long journey through milestones.

The isolation of organelles in pure populations is essential for functional analysis of the organelle-specific processes that subcellular compartments perform and understanding their cellular functions (e.g. analysis of mitochondrial electron transport, photosynthetic electron transport, CO_2_ fixation, as well as the study of transport processes). The timeline (Fig. 1) shows the long history of the development of various organelle isolation techniques in plant science, from differential centrifugation (Rho and Chipchase, 1962), through density gradient centrifugation (Miller and Romani, 1966) and free flow electrophoresis (Dubacq and Kader, 1978), to different applications using flow cytometry (Galbraith *et al.*, 1983; Harkins and Galbraith, 1987; Accotto *et al.*, 1993). Subcellular fractionation, a technique primarily employed in proteomic approaches, helps reduce the protein complexity in the sample in comparison with the total protein extract, thus revealing less abundant proteins (Huber *et al.*, 2003; Lee *et al.*, 2010). Here we report a general overview of the utilization of fluorescence-activated organelle sorting (FAOS) and highlight its potential for applications in plant sciences.

## Preparation of organelle suspension

Organelle sorting via flow cytometry can generally be applied to a wide range of plant species and tissues. Previous studies have successfully analysed and sorted organelles from diverse plant species including lycophytes (Wolf *et al.*, 2005), maize (Pfündel and Meister, 1996), grapes (Rodrigues *et al.*, 2018), Arabidopsis (Yao *et al.*, 2004), and barley (Petrovská *et al.*, 2014). Additionally, plant cell cultures can serve as suitable material for flow cytometry analysis of organelles (Rodrigues *et al.*, 2018). It is important to note that species and tissue type may influence organelle analysis parameters, such as relative size, autofluorescence background, and sorting yield.

Sample preparation is a crucial step as it significantly influences both organelle quality and data acquisition. Consistently, using fresh, healthy plant material is essential when preparing organelle suspensions. The analysis of organelles often requires their release from cells into an appropriate homogenization buffer with appropriate osmotic strength, which is critical for maintaining organelle integrity. However, plant cells are surrounded by rigid cell walls that can pose a challenge during organelle extraction. While one efficient method to rupture the cell walls is mechanical homogenization in liquid nitrogen, ice crystal formation may disintegrate organelle membranes (Song *et al.*, 2006), hindering downstream analysis. To overcome this, many protocols for plant organelle isolation start with enzymatic digestion of the cell wall. Cell wall-free protoplasts can then be gently disrupted by osmotic and/or thermal shock, causing rupturing of the plasma membrane (Somerville *et al.*, 1981; Saxena *et al.*, 1985; Robert *et al.*, 2007).

Incorporating protoplast isolation into organelle extraction can enhance the yield of intact organelles. However, the approach is time-consuming, species- and tissue-specific, and may introduce artefacts. Enzymatic digestion under high osmotic strength can trigger stress responses and alter stress-related metabolites (Flores and Galston, 1984; Gilliard *et al*., 2021). Membrane integrity may also be compromised by increasing the risk of metabolite leakage. Early studies showed that only carefully optimized preparations retained photosynthetic competence comparable with intact tissue, requiring low temperatures and short digestion times (Edwards *et al*., 1978). To mitigate these risks, monitoring aliquots at each step (tissue, protoplasts, sorted organelles, and quenched sample) helps to identify changes in target metabolites. Stable-isotope-labelled compounds can track recovery and losses, as commonly used in metabolic flux studies (Araújo *et al*., 2012; Dethloff *et al*., 2017). Leakage can also be assessed by measuring compounds in both the pellet and supernatant during a mock sorting process, as demonstrated for auxin and cytokinin analyses in protoplasts (Petersson *et al*., 2009; Antoniadi *et al*., 2015).

Alternative approaches to liquid nitrogen homogenization or protoplasting involve mechanical disruption of tissues directly in an appropriate ice-chilled homogenization buffer for organelle release (for an overview of published buffers, see Table 1; Walker, 1964). Examples include mechanical agitation through ‘bead beating’ and fine chopping using a blender or a razor blade, and tissue grinding (Leigh and Branton, 1976; Galbraith *et al.*, 1983; Keech *et al.*, 2005; Seigneurin-Berny *et al.*, 2008; Thibivilliers *et al.*, 2020). When utilizing cell cultures as a starting material, organelle release can be achieved by homogenizing cells in a glass tube with a pestle (Skalický *et al.*, 2023). This technique is both gentle in terms of organelle handling and effective, resulting in a high yield of suspended organelles.

**Table 1.** Overview of organelle isolation buffers with a focus on *Arabidopsis* species

Compartment	Buffer	pH	Osmoticum	Concentration (mM)	Reference
Nuclei	MOPS	7.0	NaCl/KCl	40/90	1
	MOPS/sodium citrate	7.0	—	—	2
	MES	5.3	Sucrose	200	3
Mitochondria	TES	8.0	Sucrose	300	4
	KH_2_PO_4_	7.3	KCl	136	5
	NH_4_HCO_3_	8.0	NaCl	200	6
Plastids	Tricine	8.4	Sorbitol	400	7
	HEPES	8.0	Sorbitol	300	8
ER	TEA-HOAc/KOAc	7.5	Sucrose	250	9
	KH_2_PO_4_	6.7	Sucrose	500	10
	Tris	7.6	NaCl	150	11
GA	Na_2_HPO_4_	—	Sucrose	400	12
Peroxisomes	Tricine	7.5	Sucrose	1000	13
Vacuoles	Na_2_HPO_4_/NaH_2_PO_4_	8.0	Mannitol	450	14
Vesicles	HEPES	7.5	Sucrose	450	15

ER, endoplasmic reticulum; GA, Golgi apparatus.

(1) Deal and Henikoff (2011); (2) Brown *et al.* (2005); (3) Skalický *et al.* (2021); (4) Boussardon *et al.* (2020); (5) Kuhnert *et al*. (2020); (6) Niehaus *et al.* (2020); (7) Seigneurin-Berny *et al.* (2008); (8) Aronsson and Jarvis (2002); (9) Kriechbaumer (2024); (10) Včelařová *et al.* (2021); (11) Wulfetange *et al.* (2011); (12) Parsons *et al.* (2012); (13) Reumann and Lisik (2017); (14) Robert *et al.* (2007); (15) Drakakaki *et al.* (2012).

Once isolated, organelles should either be fixed or promptly processed, depending on the downstream application. Removing organelles from their native intracellular conditions may induce stress and compromise their structure and functional integrity. Although the degree of fragility may vary slightly among different organelles, it is generally not recommended to store organelle suspensions for extended periods before analysis.

As a general guideline, downstream applications such as flow cytometry or omics analysis should ideally be performed within 30–60 min after organelle isolation. More delicate organelles—such as peroxisomes, chloroplasts, or mitochondria—should ideally be analysed within 15–30 min to minimize the risk of degradation or loss of function. While more robust organelles or the use of optimized suspension buffers may allow for slightly longer storage times, this must be empirically validated for each specific system. In this context, potassium-based suspension buffers are generally favoured for isolating and preserving organelles. Potassium is the predominant intracellular cation in plant cells, and its use better mimics the native ionic environment compared with sodium ions, helping to preserve organelle integrity. This is supported by the presence of organelle-localized potassium transport systems (Hamamoto and Uozumi, 2014), including tonoplast-localized TPK and NHX channels and mitochondrial K^+^ transporters, which are vital for ion homeostasis and proper organelle function (Rodríguez-Rosales *et al*., 2009; Kunz *et al*., 2014; Ahmad *et al*., 2016). Specialized metabolites that may affect the integrity and stability of the organelle(s) in suspension might be released during the isolation process. Therefore, the choice of homogenization buffer is a critical factor for obtaining reliable and high-quality organelle preparations. It should be carefully selected—and, if necessary, optimized—according to the specific organelle of interest, the plant tissue and species, the developmental stage, and the intended downstream application (Loureiro *et al*., 2021; Skalický *et al.*, 2023). For example, buffers used for chloroplast isolation often include antioxidants to preserve redox-sensitive components, while buffers for nuclei typically include both antioxidants and phenolic compound inhibitors such as polyvinylpyrrolidone (PVP) to prevent oxidation and aggregation. For downstream proteomic and lipidomic applications, the inclusion of protease and lipase inhibitors, respectively, is strongly recommended to protect analyte integrity during processing. The adverse impact of specialized metabolites on the integrity and stability of organelle(s) can be mitigated by implementing the following measures: (i) cooling of all solutions and devices used; (ii) optimizing chopping duration, technique, and intensity; and/or (iii) minimizing the time for sample preparation, flow cytometry analysis, and sorting (reviewed in Loureiro *et al.*, 2021). After obtaining an organelle suspension, additional steps such as filtration or centrifugation may be needed before injection into a cytometer to facilitate the removal of debris and/or aggregates, allowing for a faster and more straightforward analysis during flow cytometry, and thus a higher sorting speed. The complexity of organelle samples can also be reduced by fractionation, optimized for the compartment of interest. Centrifugation is a conventional, effective, and simple method for organelle enrichment (Huber *et al.*, 2003; Lee *et al.*, 2010) but could also result in yield reduction (Doležel *et al.*, 2007). Additionally, density gradient isolation of vacuoles (Rodrigues *et al.*, 2018), peroxisomes/glyoxysomes (Cooper and Beevers, 1969; Liang *et al*., 1982), or chloroplasts (Cho *et al.*, 2004) can be employed for subsequent flow cytometric analysis. A final step in sample preparation is the fine filtration of the homogenate. In this case, a pre-moistened nylon mesh, Miracloth, or other commercial filter with pore diameter ranging from 20 μm to 50 μm (also adjusted according to the plant species and the size of the organelle) is used to eliminate intact cells, large aggregates, and cellular debris (Loureiro *et al.*, 2021; Skalický *et al.*, 2023).

## Subcellular fractionation techniques

Subcellular fractionation approaches have improved to increase the resolving power and purity of isolated organelle fractions since the mid-20th century (De Duve *et al.*, 1955; Fig. 1). It is important to define the purpose of isolation (e.g. functional studies, structural analysis, and/or DNA, RNA, protein, and/or metabolites) and then compare the main pros and cons of different organelle isolation methods.

Conventional methods—such as separating organelles by sedimentation after sequential centrifugation steps (Huang and Beevers, 1971; Miflin and Beevers, 1974) or separating organelles and their inner or outer membranes via density gradient centrifugation (Soll *et al.*, 1985; Včelařová *et al*., 2021)—remain popular due to the simplicity in handling and minimal instrumentation requirements. These methods can be considered the gold standard for the preparation of crude organelle fractions or for their enrichment. Meanwhile, the demand for higher purity in isolated organelles led to the development of more specialized methods such as free-flow electrophoresis where organelles/vesicles are separated according to their overall membrane charge (Islinger *et al.*, 2018). Free-flow electrophoresis is often a step following gradient centrifugation to achieve the desired purity of the isolated organelle fraction. For instance, Golgi (Parsons *et al.*, 2012) and endoplasmic reticulum vesicles (Okekeogbu *et al.*, 2019), peroxisomes (Eubel *et al.*, 2008), and tonoplast (Bardy *et al.*, 1998) have been successfully separated for protein studies using the above-mentioned method.

Another effective organelle isolation technique utilizes affinity binding of biotin–streptavidin or epitopes to antibodies. In this approach, a specific tag/anchor is expressed in the organelle membrane of interest, while the interaction partners—streptavidin or antibody—are immobilized on the surface of magnetic particles. Therefore, the addition of these magnetic particles to the cell homogenate allows selective capture and isolation of the desired organelles. This method has been firstly applied to isolation of nuclei (known as INTACT) from root cells (Deal and Henikoff, 2010, 2011). Recently, a derivative of this method has been published for mitochondria and plastid isolation called IMTACT and IPTACT, respectively (Boussardon *et al.*, 2020; Boussardon and Keech, 2023). Moreover, antibody-coupled beads have been successfully used for purification of post-Golgi transport vesicles (Wilkop *et al.*, 2019). The affinity purification method provides cell-specific resolution depending on tag expression and high purity as great advantages. However, affinity purification requires time-consuming generation of transgenic plant lines or antibody preparation.

Flow cytometry is a well-established technique in the mammalian field, and one of the first applications was bacterial detection and blood cell analysis (summarized in Shapiro, 2024). Later, flow cytometry was widely utilized in immunology (Cossarizza *et al*., 2021), microbiology (Steen, 2000), and for plant cell/protoplast isolation (Rogers *et al*., 2012; Carter *et al.*, 2013; Antoniadi *et al*., 2022). This technique has also been applied for sorting of organelles, such as nuclei or chloroplasts (Pfündel and Meister, 1996; Šafář *et al.*, 2004; Wolf *et al.*, 2005; Petrovská *et al.*, 2014; Haas *et al.*, 2016; Gaiero *et al.*, 2018; Gutzat *et al.*, 2020; Thibivilliers *et al.*, 2020). FAOS can facilitate simultaneous sorting of more than one organelle population from the same plant material while monitoring changes in live organelle conditions. Moreover, flow cytometry not only has cell-specific resolution but can also empower distinction between nuclei in different stages of the cell cycle (Petrovská *et al.*, 2014; Haas *et al.*, 2016; Chamrád *et al.*, 2018). Further details on isolation of nuclei by FAOS can be found in Loureiro *et al*. (2021). FAOS also allows for subcellular fractionation while in parallel enabling the analysis of additional parameters, as discussed later in this review. All mentioned fractionation/isolation methods are summarized in Table 2.

**Table 2.** Overview of published isolation methods for a particular organelle

Compartment	Isolation methods				
Nuclei	DC (1, 2)	GC (3)		AP (22, 23)	FCM (29–36)
Nucleoli	DC (2)				
Vacuoles		GC (4, 5)	FFE (17)		
Plastids		GC (6–8)		AP (24)	FCM (36–38)
Mitochondria		GC (9)	FFE (18)	AP (25–27)	
ER		GC (10–13)	FFE (19)		FCM (36)
GA		GC (14)	FFE (20)	AP (28)	
Peroxisomes		GC (15,16)	FFE (21)		

AP, affinity purification; DC, differential centrifugation; ER, endoplasmic reticulum; FCM, flow cytometry; FFE, free-flow electrophoresis; GA, Golgi apparatus.

(1) Saxena *et al*. (1985); (2) McKeown *et al*. (2008); (3) Folta and Kaufman (2006); (4) Saunders (1979); (5) Robert *et al*. (2007); (6) Somerville *et al*. (1981); (7) Aronsson and Jarvis (2002); (8) Seigneurin-Berny *et al.* (2008); (9) Keech *et al.* (2005); (10) Chen *et al.* (2002); (11) Wulfetange *et al.* (2011); (12) Ding *et al.* (2012); (13) Včelařová *et al.* (2021); (14) Muñoz *et al.* (1996); (15) Reumann and Lisik (2017); (16) Huang and Beevers (1971); (17) Bardy *et al.* (1998); (18) Eubel *et al.* (2007); (19) Okekeogbu *et al.* (2019); (20) Parsons *et al.* (2012); (21) Eubel *et al.* (2008); (22) Deal and Henikoff (2011); (23) Moreno-Romero *et al.* (2017); (24) Boussardon and Keech (2023); (25) Boussardon *et al.* (2020); (26) Kuhnert *et al.* (2020); (27) Niehaus *et al.* (2020); (28) Wilkop *et al.* (2019); (29) Petrovská *et al.* (2014); (30) Haas *et al.* (2016); (31) Chamrád *et al.* (2018); (32) Gutzat *et al.* (2020); (33) Thibivilliers *et al.* (2020); (34) Loureiro *et al.* (2021); (35) Gaiero *et al.* (2018); (36) Skalický *et al.* (2023); (37) Pfündel and Meister (1996); (38) Wolf *et al.* (2005).

Overall, different isolation techniques can result in varying degrees of organelle enrichment and purity. Thus, it is essential to verify the quality of isolated fractions using enzymatic activity assays, western blotting, or proteomic analysis to quantify the extent of contamination from other organelles, cytosolic components, or material adhering to the organelle surface (Stitt *et al.*, 1989; Fürtauer *et al.*, 2016; Boussardon *et al.*, 2020; Skalický *et al.*, 2023). A set of marker enzymes or proteins should be used to assess cross-contamination of isolated organelles, ensuring that markers are detectable with sufficient sensitivity to quantify even low levels of contaminating organelles. Quantification should be related back to the total tissue or protoplast/organelle suspension, which can be achieved through enzymatic activity measurements, providing semi-quantitative information (Stitt *et al.*, 1989; Lee *et al.*, 2010). While contamination after FAOS may be lower than with other isolation methods, systematic comparisons are lacking and represent an important area for future investigation. If the goal is to study organelle-specific mechanisms—such as metabolic events, their regulation, or coupling of oxidative phosphorylation—assessment of functional competence is also necessary.

It is important to keep in mind that metabolite content may change during isolation due to rapid metabolic turnover unless the plant material is frozen or enzymatic activity is halted, as in non-aqueous fractionation. (Stitt *et al.*, 1989; Fürtauer *et al*., 2016). When these conditions cannot be maintained due to downstream application requirements, as in FAOS, control experiments are essential. One strategy is to analyse aliquots at each step of the FAOS workflow or to use stable-isotope-labelled compounds to monitor potential changes in target metabolites (Skalický *et al.*, 2023). In addition to these quantitative assessments, purity and functional competence can be rigorously evaluated post-sort using fluorescent organelle markers or dyes (Skalický *et al.*, 2021) in combination with validation methods, such as enzymatic activity assays or proteomic analysis. For example, mitochondria sorted via genetically encoded fluorescent proteins can be tested for both post-sort fluorescence and respiration, while chloroplasts can be validated through chlorophyll autofluorescence and enzymatic assays. Incorporating such controls ensures that FAOS-derived organelles are both sufficiently pure and functionally relevant for downstream applications.

## Effective organelle identification using flow cytometric techniques

Although homogenization techniques can produce high yields of organelle release, organelle suspension mixtures usually contain a large number of different particles (Seigneurin-Berny *et al.*, 2008). These refer to different types of undesirable material such as broken or impaired organelles, fragments of undigested cell walls, or other remnants of cellular debris that could not be removed during the sample preparation procedure. Therefore, an organelle suspension is often a highly heterogeneous mixture.

**Fig. 2.**
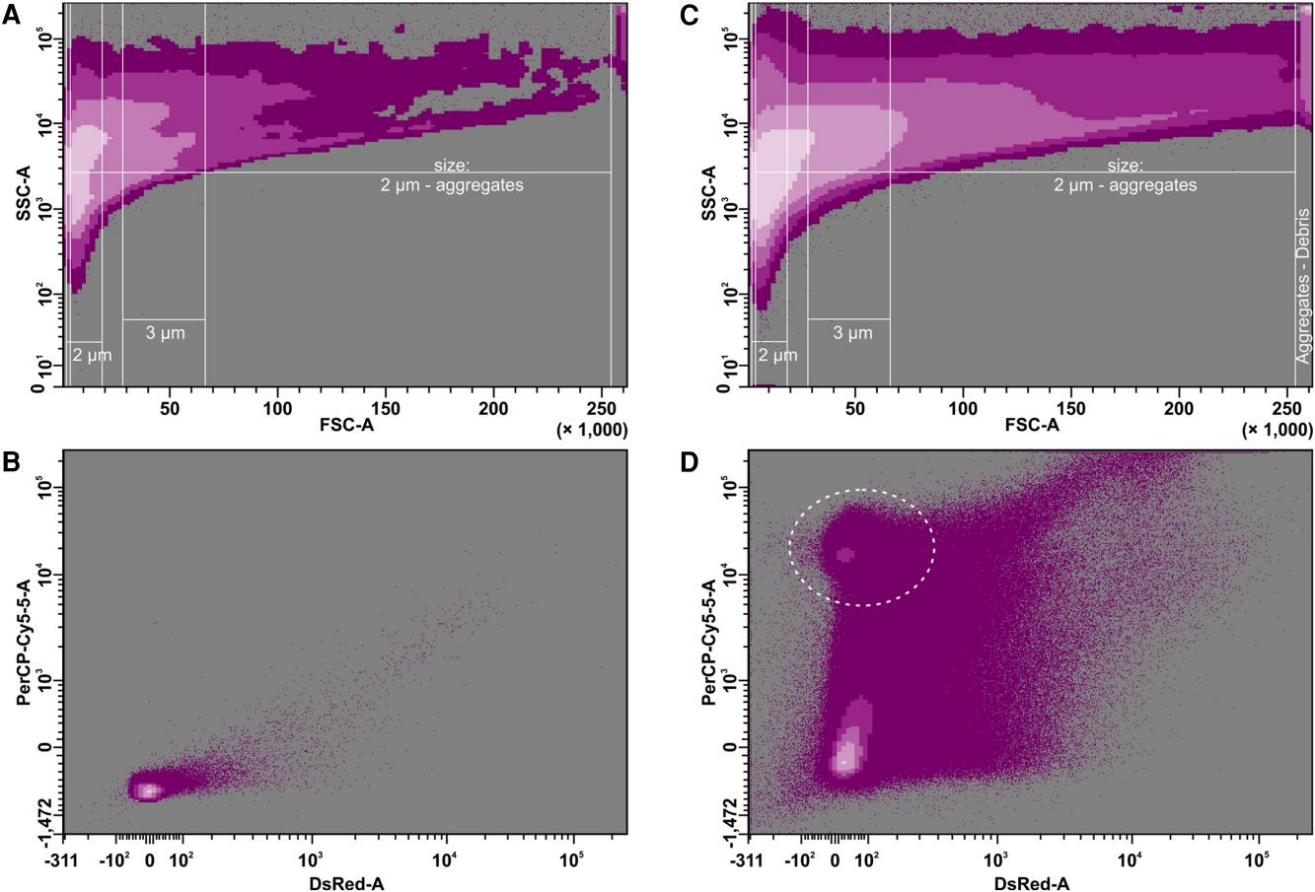
Representative output of flow cytometric analysis of a cellular homogenate of *Arabidopsis thaliana.* The illustrative figure derives from the dataset of Skalický *et al.* (2023) showing an etiolated cell culture (A, B) and a culture cultivated under continuous light (C, D). (A and C) Organelle density distribution based on FSC (forward-scattered light) versus SSC (side-scattered light). The FSC of commercially available bead populations with known diameters (2 μm and 3 μm) is defined by the respective gates, as shown. Therefore, the population of organelles lies between the relative size of 2 μm and the size of aggregates on the forward scatter axis forming a large population located empirically at the right top edge of the chart, as indicated. (B and D) Organelle density distribution based on the organelle suspension autofluorescence. Biplots based on the fluorescence scatter perceived by PerCP-Cy5-5 (695/40 nm) and DsRed (582/15 nm) filters after excitation with a blue (488 nm) and yellow-green (561 nm) laser, respectively. In (D), the core of the chloroplast population, with strong autofluorescence perceived by PerCP-Cy5-5, is indicated by a bordered circle.

Previous cytological studies (Wada and Suetsugu, 2004; Sharma *et al*., 2018; Skalický *et al*., 2023) report that the size of *Arabidopsis thaliana* organelles typically ranges from submicron dimensions, as in peroxisomes and small vesicles (0.2–1 μm), to several microns, such as in chloroplasts (5–7 μm) and nuclei (5–10 μm), depending on organelle type, developmental stage, and cellular context. As this size range can also include various types of debris, clearly identifying organelle populations solely by their relative size or granularity/complexity parameters via flow cytometry (forward and side scatter biplot, Fig. 2A, C) is challenging. Moreover, organelle sizes and shapes may slightly differ depending on the cell type, developmental stage, and the plant species under study (Dittmer *et al.*, 2007; Oda and Fukuda, 2011; Boussardon *et al.*, 2020). However, analysis of these parameters (relative size, granularity, and complexity) by flow cytometry can exclude large amounts of debris. Thus, populations of very small particles/compartments (<2 µm) and large particles or aggregates (>10–20 µm) can be omitted from the analysis. It should also be mentioned that the forward and side scatter signals are empirically derived and do not depict the absolute size and granularity/complexity of the analysed particles. By representing the forward and side scatter of calibration beads with already known diameters (Fig. 2A, B), it is possible to obtain an estimate of relevant particle sizes compared with the beads. After defining the relative size range of organelles, a more precise analysis can be conducted based on the specific fluorescence parameters of the organelles.

Fluorescence can be exploited as a key feature for organelle population identification and analysis by flow cytometry. Specific localization of potential fluorochromes is required. Plant cells and organelles contain a wide spectrum of autofluorescent compounds, such as chlorophyll, flavins, lignin, and advanced glycation end-products (summarized in Antoniadi *et al.*, 2022), which can make the analysis more challenging. On the other hand, strong chlorophyll autofluorescence, occurring only in chloroplasts, may facilitate their identification (Fig. 2B, D).

**Fig. 3.**
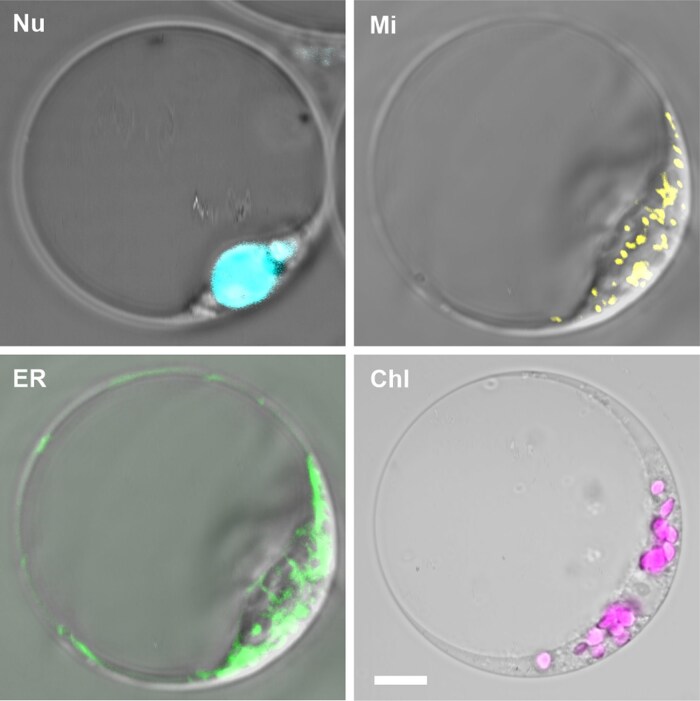
Subcellular compartment staining. Representative photos of individual cells stained with different dyes (Skalický *et al.*, 2023). Nuclei (Nu) stained with Hoechst 33342, mitochondria (Mi) with MitoTracker Orange, endoplasmic reticulum (ER) with ER-Tracker Green, and chloroplasts (Chl) visualised through chlorophyll autofluorescence. Scale bars are 5 µm.

An alternative method for determining a population involves fluorescent staining. While the application of fluorescently labelled antibodies against plant proteins is impractical due to limited commercial availability and usage, a variety of organelle-specific fluorescent dyes serve as viable alternatives (Fig. 3). Commercially available fluorescent dyes (MitoTracker, ER tracker, Hoechst, and DAPI; Table 2) are effective in staining the majority of plant organelles and have already found applications in plant sciences (Skalický *et al.*, 2023), although they were initially designed for mammalian cells (Cho *et al.*, 2004; Yao *et al.*, 2004; Wolf *et al.*, 2005; Petrovská *et al.*, 2014). However, the original design of these dyes may necessitate additional testing before use, as certain aspects of plant and animal cells differ, such as cell permeability and dye specificity. In general, fluorescent dyes can be categorized into two groups according to whether they are used to stain live or fixed samples, with some dyes accommodating both sample preparation variants. A major advantage of the fluorescent dyes is that they can be applied in all genotypes (e.g. mutant and overexpressing lines) as well as in different species, making them a precious and efficient tool in genetic and functional studies. Moreover, one sample can be stained with a mixture of several fluorescent dyes, as shown previously by the application of ER-Tracker Green, MitoTracker Orange, and Hoechst 33342 to organelles derived from a 14-day-old cell culture (Skalický *et al.*, 2023; Fig. 3).

Finally, the use of transgenic plant lines expressing organelle-specific fluorescent markers is fundamental for accurate and clear identification of organelle populations. These plant lines have been engineered to express a fluorescent protein in fusion with a targeting sequence or protein that specifically localizes to a certain organelle. For this approach, it is important to select marker proteins/sequences that display a unique subcellular localization. Due to the pre-existing autofluorescence of the plant cell, addition of an engineered fluorescent protein (or fluorescent dye) may result in a population shift in terms of fluorescence. For this reason, it is critical to always analyse good quality controls (e.g. wild-type plant cells and/or unstained samples) and subsequently adjust the flow cytometer analysis.

Organelle-specific fluorescence is generally required to clearly identify an organelle population of interest via FAOS (Tables 3, 4; Fig. 3). The relevant FAOS gates can then be determined and the organelles can be analysed and/or sorted. For sorting and integrity maintenance of non-fixed organelles, it is recommended to keep the loading and collection chamber of the cytometer at 4 °C and to add a small amount of an appropriate buffer to the pre-cooled collection tube depending on the downstream analysis. FAOS is a powerful approach to subcellular fractionation, and more efficient protocols for routine organelle sorting will need to be developed in the future. Recently, several basic protocols for sorting of nuclei including best practices have been published (Chamrád *et al.*, 2018; Thibivilliers *et al.*, 2020; Loureiro *et al.*, 2021).

**Table 3.** Overview of representative commercially available fluorescent dyes for organelle staining

Compartment	Fluorescent dye	Wavelength (nm)	
		Excitation (laser)	Emission (colour)
Nuclei	DAPI (1)	360 (UV)	460 (violet/blue)
	Hoechst 33342 (2)	361 (UV)	497 (violet/blue)
	Sytox™ Green	504 (blue)	523 (green)
	Propidium iodide (3)	532 (green)	617 (orange)
Mitochondria	MitoTracker™ Green FM (4, 5)	490 (blue)	516 (green)
	MitoTracker™ Orange CMTMRos (6)	554 (green-yellow)	576 (yellow)
	MitoTracker™ Red FM (7)	581 (green-orange)	644 (red)
	JC-1	498/593 (blue/yellow)	525/595 (green/orange)
	MitoTracker™ Deep Red FM	644 (red)	665 (red)
ER	ER-Tracker™ Blue-White DPX (8)	374 (UV)	430–640 (violet-red)
	ER-Tracker™ Green (9)	502 (blue)	511 (green)
	ER-Tracker™ Red (10,11)	588 (yellow)	615 (orange-red)
GA	BODYPI™ FL C_5_-Ceramide	502 (blue)	511 (green)
	NBD C_6_-Ceramide	466 (blue)	536 (green-yellow)
	BODYPI™ TR Ceramide (8)	588 (yellow)	615 (orange-red)

ER, endoplasmic reticulum; GA, Golgi apparatus.

(1) Petrovská *et al.* (2014); (2) Haas *et al.* (2016); (3) Thibivilliers *et al.* (2020); (4) Cho *et al.* (2004); (5) Wolf *et al.* (2005); (6) Boussardon *et al.* (2020); (7) Yao *et al.* (2004); (8) Wang *et al.* (2015); (9) Skalický *et al.* (2023); (10) Hayashi *et al.* (2014); (11) Zalabák *et al.* (2016).

**Table 4.** Overview of organelle-specific markers fused with fluorescent proteins

Compartment	Marker protein/sequence	Shortcut	Reference
Nuclei	Histone 2B	H2B	Boisnard-Lorig *et al.* (2001)
Mitochondria	β-ATPase	β-ATPase	Logan and Leaver (2000)
Plastids	Snowy cotyledon 1	SCO1	Albrecht *et al.* (2006)
ER	HDEL anchoring motif	HDEL	Matsushima *et al.* (2002)
ER	KDEL anchoring motif	KDEL	Matsushima *et al.* (2002)
GA	*N*-Acetylglucosaminyl transferase I	NAG1	
Peroxisomes	PTS1 sequence	PTS1	Grebe *et al.* (2003)
Tonoplast	V-type proton ATPase subunit a3	VHA-a3	Nelson *et al.* (2007)
Vacuoles	Aquaporins	TIPs	Viotti *et al.* (2013)
Lytic vacuoles	Aleurain	Aleurain	Ma *et al.* (2004)
Storage vacuoles	Chitinase	Chi	Miao *et al.* (2008)
Early endosomes	Ras-related protein RABA2a	Rab-A2	Fluckiger *et al.* (2003)
Vesicles	Clathrin	CLC	Markham *et al*. (2011)

ER, endoplasmic reticulum; GA, Golgi apparatus.

## Plant organelle sorting in various applications

FAOS offers a wide spectrum of applications, allowing the measurement of various parameters during analysis. It facilitates the examination and sorting of thousands of organelles in a short time, generating extensive datasets based on the light scattering characteristics of the organelles suitable for robust statistical analysis of the populations of interest. The resulting data can be used for structural and functional studies of organelles, as previously reported for yeasts (Rodrigues *et al.*, 2013, 2018).

A key feature of isolated organelles is their integrity, which can be evaluated by FAOS analysis. The relative size and shape (granularity/complexity parameters) of each organelle can be continuously monitored during cytometric analysis. Thus, if there are any dramatic changes in these characteristics such as when an organelle breaks, it is automatically excluded from the gate on the biplot of forward and side scatter (Fig. 2A) and thus it is neither sorted nor included in the analysis. However, the integrity and functionality of an organelle require more precise analyses. Several commercially available kits can test this by measuring the metabolism or uptake of a fluorescent dye by healthy and functional organelles, such as carboxyfluorescein diacetate for the determination of chloroplast integrity and viability (Schulz *et al.*, 2004). Organelle functionality can also be analysed on a single-cell basis in protoplasts. Yao and co-workers have developed an FAOS technique to quantitate changes in mitochondrial membrane potential during programmed cell death (Yao *et al.*, 2004).

Once the subcellular organelle of interest has been isolated and/or the respective analytical data have been gathered, numerous downstream applications can take place. The minimum amount of sorted material required depends primarily on the intended application—specifically, the sensitivity of the instrumentation, the abundance and stability of the target molecules within the organelles, and the type of organelle being analysed. Currently, one of the most prominent cytometry applications involving subcellular compartments in plant science is the sorting of nuclei or chromosomes for DNA sequencing in species with large and complex genomes. A notable example is wheat (*Triticum aestivum*), which has a highly repetitive, allohexaploid genome. In this context, flow-sorted chromosomes have greatly facilitated the assembly and annotation of individual chromosomes (Šimková *et al.*, 2003; International Wheat Genome Sequencing Consortium, 2018), making it a benchmark case for the utility of cytometric sorting in genomics. Thousands to tens of thousands of nuclei have also been sorted for bulk or single-nuclei transcriptome analysis, respectively (Gutzat *et al.*, 2020; Thibivilliers *et al.*, 2020; Farmer *et al.*, 2021). Moreover, FAOS has been exploited as a tool for semi-autonomous organelle isolation for subsequent chloroplast genome sequencing (Wolf *et al.*, 2005) and non-nuclear genome characterization (Cho *et al.*, 2004).

Proteomics is another research field that benefits from the FAOS technique. Given the high complexity of the plant cell proteome, methods such as 2D electrophoresis or other types of fractionation (Huber *et al.*, 2003) have been necessary to enhance protein separation. However, the detection of abundant proteins can overshadow the identification of low-abundance proteins, thus reducing the overall comprehensiveness of protein identification (Lee *et al.*, 2010). As a result, subcellular fractionation methods based on (ultra)centrifugation have been employed. Nonetheless, the resolving power of these conventional methods may prove insufficient. In contrast, the FAOS technique offers highly accurate sample analysis and fractionation. Proteomic studies of nuclear proteins in barley requiring ∼5 million nuclei have shown that FAOS not only enables the separation of organelles but also facilitates the monitoring of organelles at various developmental stages (Petrovská *et al.*, 2014; Blavet *et al.*, 2017). Although protocols for proteomic studies of mammalian organelles are well established (Godfrey *et al*., 2005), applications of these techniques in the plant field are still limited.

Recently, a FAOS-based protocol was developed for the simultaneous sorting of nuclei, endoplasmic reticulum, chloroplasts, and mitochondria from the same sample (Skalický *et al*., 2023), facilitating plant hormone metabolite quantification and protein identification within the cell compartments. These findings collectively demonstrate that FAOS can be utilized for genomic, transcriptomic, proteomic, and metabolomic studies of plant organelles. Furthermore, FAOS could also be leveraged for more complex ‘omic’ studies at the subcellular level, addressing a challenge posed by other isolation protocols, which often isolate only one type of organelle per sample. Following the selection of desired organelle populations, many instruments can perform simultaneous four- to six-way sorting of different populations, increasing both efficiency and comparability of results. Using identical sample handling for all organelles further improves the reliability of comparisons, offering an advantage over methods such as differential centrifugation and/or density gradient ultracentrifugation.

Both the injected sample and the sorted organelles in the flow cytometer are maintained at 4 °C, minimizing metabolic and enzymatic activity, reducing degradation, and preserving organelle integrity throughout the sorting process. However, certain analytes may remain sensitive and susceptible to degradation after prolonged exposure (several hours)—even at this low temperature. Therefore, the appropriate collection duration (e.g. 20–30 min) should be determined based on the stability of the analytes and the requirements of the downstream application. Pooling samples after multiple short sorting sessions is a practical strategy to preserve sample quality while achieving the desired quantity of organelles for subsequent analyses. Notably, it has been shown that profiles of phytohormones—which are molecules with highly dynamic turnover—remain largely stable during a 30 min sorting period (Skalický *et al*., 2023).

## Biological advancements facilitated by organelle sorting

Over the years, the isolation of plant organelles has helped address some of the most fundamental questions in plant biology. By studying compartments such as chloroplasts and mitochondria, researchers have identified where key metabolic processes—such as photosynthesis and respiration—occur. The regulation of these processes has been investigated using isolated functional organelles, which would not be possible in whole tissue. Moreover, organelle isolation has been essential for elucidating and characterizing intracellular transporters (Piechulla and Heldt, 2025). Organelle isolation has enabled the mapping of proteins and transcripts to subcellular locations, revealing how gene expression is coordinated across compartments (van Wijk and Baginsky, 2011; Woodson and Chory, 2008). Isolated peroxisomes have been pivotal in elucidating how plants detoxify reactive oxygen species (ROS) and manage ion homeostasis under stress (Reumann *et al.*, 2007; Hu *et al.*, 2012), while nuclear isolations have allowed genome-wide studies of chromatin structure, transcriptional regulation, and epigenetic control (reviewed in Dvořáčková *et al.*, 2024). The isolation of vacuoles has also shed light on peptide import, RNA degradation, and membrane transporter function, enhancing our understanding of vacuolar trafficking and cellular recycling.

More recently, the advent of FAOS and its variants has opened up new avenues for high-resolution studies of organelle-specific processes. For instance, the INTACT method (Deal and Henikoff, 2011) enabled cell-type-specific chromatin and transcriptomic profiling through the fluorescent tagging and isolation of nuclei in *A. thaliana*, deepening our understanding of gene regulation and epigenetic landscapes during plant development. Petrovská *et al.* (2014) applied FAOS to analyse the nuclear proteome during specific cell cycle phases in barley, advancing knowledge of nuclear dynamics in plants. Organelle sorting has also enabled unprecedented resolution in plant genomics and transcriptomics, facilitating the assembly and annotation of complex genomes and supporting bulk and single-nucleus transcriptome analyses, and allowing precise isolation of organelles for chloroplasts and characterization of other non-nuclear genomes (Šimková *et al.*, 2003; International Wheat Genome Sequencing Consortium, 2018; Gutzat *et al.*, 2020; Thibivilliers *et al.*, 2020; Farmer *et al.*, 2021).

FAOS has further extended beyond nuclear studies. Using the technique, Wolf *et al.* (2005) generated the first complete chloroplast genome of a lycophyte, providing critical insights into the early evolution of vascular plants, clarifying the ancestral chloroplast DNA structure and gene content, and enabling comparative genomics to trace the diversification of photosynthetic pathways, genome organization, and adaptive strategies across plant lineages. Moreover, FAOS facilitated the isolation of chloroplasts and mitochondria from *Nicotiana benthamiana*, revealing that DNA gyrase activity influences organelle morphology, DNA content, and nucleoid structure. This work uncovered a previously unappreciated mechanism by which DNA topology regulates organelle genome organization and function in plants (Cho *et al.,* 2004).

Understanding intracellular hormone regulation—supported by evidence from hormone-related protein localization (reviewed by Skalický *et al*., 2018 )—is also essential, as plant hormones orchestrate growth, development, and environmental responses. Determining how hormones are regulated within specific organelles or compartments provides insight into the precise mechanisms that plants use to fine-tune these processes and adapt to changing conditions. In this context, FAOS-isolated nuclei from *A. thaliana* revealed dynamic auxin homeostasis in response to precursor and hormone treatments (Skalický *et al.*, 2021). Building on this, a methodological advance by Skalický *et al.* (2023) enabled simultaneous profiling of auxin and cytokinin metabolites across four organelles, revealing distinct and dynamic hormone distribution patterns and highlighting the power of FAOS to dissect organelle-specific hormone regulation.

Altogether, organelle sorting has not only increased the resolution at which plant cells can be studied but has also exposed new dimensions of organelle communication and adaptation to developmental and environmental cues, establishing it as a transformative tool for dissecting plant cell function and subcellular complexity.

## Future technical and biological perspectives

FAOS is emerging as a transformative tool in plant biology, enabling the precise isolation and analysis of intact organelles from heterogeneous tissues. Its flexibility, throughput, and quantitative capabilities make it ideally suited to address fundamental and applied biological questions with unprecedented resolution.

FAOS offers single-organelle sensitivity and is compatible with a broad array of fluorescent dyes and genetically encoded markers (Tables 3, 4), allowing for organelle-specific labelling tailored to experimental needs (Fig. 3). Real-time, live-cell organelle analysis preserves physiological states during sorting, ensuring that downstream applications are based on biologically relevant material. The recent development of multi-organelle sorting (Skalický *et al*., 2023) further extends this potential by enabling the simultaneous analysis of multiple organelles from the same sample. Through integration with image-enabled and spectral cytometry, it is now possible to perform high content analysis based on organelle morphology, size, and dynamics. (Nitta *et al*., 2018; Schraivogel *et al*., 2022).

FAOS provides an opportunity to dissect organelle-specific functions during complex biological processes. For example, vacuole sorting could reveal mechanisms of RNA and peptide degradation, storage, or hormone compartmentalization—particularly relevant for understanding auxin and cytokinin metabolism in root development. Peroxisome isolation could be used to monitor ROS buffering capacity under salt or drought stress and to investigate peroxisomal contributions to β-oxidation and jasmonate biosynthesis (Reumann *et al*., 2007). Nuclear and nucleolar sorting can support genome-wide analysis of chromatin and rRNA biogenesis, respectively, allowing studies of transcriptional reprogramming or stress-induced nucleolar remodelling in hormone or nutrient signalling pathways (Shav-Tal *et al*., 2005).

FAOS can be instrumental in understanding how developmental transitions are coordinated at the organelle level. For instance, during lateral root initiation, plastids, mitochondria, and vacuoles undergo structural and metabolic shifts; isolating these organelles from specific developmental stages or cell types could reveal organelle-autonomous cues for reprogramming. Combined with lineage tracing and reporter-based labelling, FAOS can enable spatially and temporally resolved organelle profiling to uncover compartment-specific regulators of cell fate acquisition and differentiation.

Coupling FAOS with proteomics, transcriptomics, metabolomics, and lipidomics provides access to the molecular cargo of individual organelles under specific conditions. For example, nucleus- or mitochondrion-targeted FAOS followed by RNA sequencing or MS can identify compartment-localized transcripts or proteins involved in stress memory, light signalling, or energy metabolism. Additionally, FAOS combined with MS is a suitable technique for studying organelle membrane lipid composition and its modification and/or reorganization in response to different stress conditions (Garcia-Hernandez *et al*., 2025). Top-down proteomics applied to sorted chloroplasts could uncover photosystem remodelling under fluctuating light, while vacuolar metabolomics could identify novel conjugated phytohormones or specialized metabolites. The application of single-organelle multi-omics is an emerging frontier that FAOS is uniquely positioned to support.

FAOS can play a critical role in testing and optimizing synthetic biology strategies. Organelles serve as specialized biochemical reactors, and sorting transformed organelles allows rapid assessment of synthetic pathway performance *in vivo*. For instance, mitochondria engineered to express alternative respiratory components or plastids modified to host heterologous metabolic pathways can be isolated and assayed directly, without the need for whole-tissue extraction. FAOS also enables screening of genetically encoded biosensors targeted to specific organelles, accelerating the design–build–test cycles in organelle engineering.

Age-related functional decline of organelles such as chloroplasts, mitochondria, and peroxisomes impacts plant senescence, productivity, and stress resilience. FAOS could facilitate time-course studies of organelle turnover, allowing researchers to capture ‘aged’ organelle populations and characterize their proteomic or metabolic deterioration. For example, changes in vacuolar pH or protease content during senescence could be profiled directly from sorted vacuoles. Similarly, isolation of mitochondria from ageing root tips could help uncover mechanisms of respiratory compensation or decline.

FAOS can be applied across species to investigate the conservation and diversification of organelle function. Comparative studies between Arabidopsis, crop species, and basal land plants could identify evolutionary innovations in organelle content, biogenesis, and interaction. For instance, FAOS of plastids in C_3_ versus C_4_ plants might cast light on adaptations in photosynthetic efficiency, while nucleolar sorting across species may uncover differences in rRNA regulation linked to growth rate or stress tolerance.

## Conclusion

In summary, FAOS and its evolving variants are poised to become foundational tools in plant biology, enabling the isolation and analysis of organelles with cell type and condition specificity. By bridging cytometry, imaging, and multi-omics technologies—including transcriptomics, proteomics, metabolomics, lipidomics, and hormonomics—FAOS offers unprecedented access to the inner workings of subcellular compartments. This will open up new avenues to study developmental reprogramming, organelle-specific signalling, stress adaptation, ageing, and evolution. Its precision and scalability make FAOS particularly well suited to uncover organelle contributions to cellular homeostasis, interorganelle and intercellular communication, and plant–pathogen interactions. As instrumentation and labelling strategies advance, FAOS is expected to contribute significantly to a holistic and dynamic understanding of plant cell organization, function, and metabolic regulation.

## References

[eraf490-B1] Accotto GP, Mullineaux PM, Brown SC, Marie D. 1993. Digitaria streak geminivirus replicative forms are abundant in S-phase nuclei of infected cells. Virology 195, 257–259. 8317101 10.1006/viro.1993.1369

[eraf490-B2] Ahmad K, Waris M, Hayat M. 2016. Prediction of protein submitochondrial locations by incorporating dipeptide composition into Chou’s general pseudo amino acid composition. Journal of Membrane Biology 249, 293–304. 26746980 10.1007/s00232-015-9868-8

[eraf490-B3] Albrecht V, Ingenfeld A, Apel K. 2006. Characterization of the *snowy cotyledon 1* mutant of *Arabidopsis thaliana*: the impact of chloroplast elongation factor G on chloroplast development and plant vitality. Plant Molecular Biology 60, 507–518. 16525888 10.1007/s11103-005-4921-0

[eraf490-B4] Antoniadi I, Plačková L, Simonovik B, Doležal K, Turnbull C, Ljung K, Novák O. 2015. Cell-type-specific cytokinin distribution within the Arabidopsis primary root apex. The Plant Cell 27, 1955–1967. 26152699 10.1105/tpc.15.00176 PMC4531351

[eraf490-B5] Antoniadi I, Skalický V, Sun G, Ma W, Galbraith DW, Novák O, Ljung K. 2022. Fluorescence activated cell sorting—a selective tool for plant cell isolation and analysis. Cytometry Part A 101, 725–736. 10.1002/cyto.a.24461 34028996

[eraf490-B6] Araújo WL, Nunes-Nesi A, Nikoloski Z, Sweetlove LJ, Fernie AR. 2012. Metabolic control and regulation of the tricarboxylic acid cycle in photosynthetic and heterotrophic plant tissues. Plant, Cell & Environment 35, 1–21. 10.1111/j.1365-3040.2011.02332.x 21477125

[eraf490-B7] Aronsson H, Jarvis P. 2002. A simple method for isolating import-competent *Arabidopsis* chloroplasts. FEBS Letters 529, 215–220. 12372603 10.1016/s0014-5793(02)03342-2

[eraf490-B8] Bardy N, Carrasco A, Galaud JP, Pont-Lezica R, Canut H. 1998. Free-flow electrophoresis for fractionation of *Arabidopsis thaliana* membranes. Electrophoresis 19, 1145–1153. 9662177 10.1002/elps.1150190715

[eraf490-B9] Blavet N, Uřinovská J, Jeřábková H, Chamrád I, Vrána J, Lenobel R, Beinhauer J, Šebela M, Doležel J, Petrovská B. 2017. UNcleProt (universal nuclear protein database of barley): the first nuclear protein database that distinguishes proteins from different phases of the cell cycle. Nucleus 8, 70–80. 27813701 10.1080/19491034.2016.1255391 PMC5287097

[eraf490-B10] Boisnard-Lorig C, Colon-Carmona A, Bauch M, Hodge S, Doerner P, Bancharel E, Dumas C, Haseloff J, Berger F. 2001. Dynamic analyses of the expression of the HISTONE::YFP fusion protein in Arabidopsis show that syncytial endosperm is divided in mitotic domains. The Plant Cell 13, 495–509. 11251092 10.1105/tpc.13.3.495 PMC135513

[eraf490-B11] Boussardon C, Keech O. 2023. Tissue-specific isolation of tagged *Arabidopsis* plastids. Current Protocols 3, e673. 36799650 10.1002/cpz1.673

[eraf490-B12] Boussardon C, Przybyla-Toscano J, Carrie C, Keech O. 2020. Tissue-specific isolation of Arabidopsis/plant mitochondria—IMTACT (isolation of mitochondria tagged in specific cell types). Plant Journal 103, 459–473. 10.1111/tpj.14723 32057155

[eraf490-B13] Brown JK, Lambert GM, Ghanim M, Czosnek H, Galbraith DW. 2005. Nuclear DNA content of the whitefly *Bemisia tabaci* (Aleyrodidae: Hemiptera) estimated by flow cytometry. Bulletin of Entomological Research 95, 309–312. 16048678 10.1079/ber2005361

[eraf490-B14] Carter AD, Bonyadi R, Gifford ML. 2013. The use of fluorescence-activated cell sorting in studying plant development and environmental responses. International Journal of Developmental Biology 57, 545–552. 24166437 10.1387/ijdb.130195mg

[eraf490-B15] Chamrád I, Uřinovská J, Petrovská B, Jeřábková H, Lenobel R, Vrána J, Doležel J, Šebela M. 2018. Identification of plant nuclear proteins based on a combination of flow sorting, SDS–PAGE, and LC-MS/MS analysis. Methods in Molecular Biology 1696, 57–79. 29086396 10.1007/978-1-4939-7411-5_4

[eraf490-B16] Chen YF, Randlett MD, Findell JL, Schaller GE. 2002. Localization of the ethylene receptor ETR1 to the endoplasmic reticulum of Arabidopsis. Journal of Biological Chemistry 277, 19861–19866. 11916973 10.1074/jbc.M201286200

[eraf490-B17] Cho HS, Lee SS, Kim KD, Hwang I, Lim JS, Park YI, Pai HS. 2004. DNA gyrase is involved in chloroplast nucleoid partitioning. The Plant Cell 16, 2665–2682. 15367714 10.1105/tpc.104.024281 PMC520963

[eraf490-B18] Cooper TG, Beevers H. 1969. Mitochondria and glyoxysomes from castor bean endosperm. Enzyme constitutents and catalytic capacity. Journal of Biological Chemistry 244, 3507–3513. 5798623

[eraf490-B19] Cossarizza A, Chang HD, Radbruch A, et al 2021. Guidelines for the use of flow cytometry and cell sorting in immunological studies (third edition). European Journal of Immunology 51, 2708–3145. 34910301 10.1002/eji.202170126 PMC11115438

[eraf490-B20] Deal RB, Henikoff S. 2010. A simple method for gene expression and chromatin profiling of individual cell types within a tissue. Developmental Cell 18, 1030–1040. 20627084 10.1016/j.devcel.2010.05.013 PMC2905389

[eraf490-B21] Deal RB, Henikoff S. 2011. The INTACT method for cell type-specific gene expression and chromatin profiling in *Arabidopsis thaliana*. Nature Protocols 6, 56–68. 21212783 10.1038/nprot.2010.175 PMC7219316

[eraf490-B22] De Duve C, Pressman BC, Gianetto R, Wattiaux R, Appelmans F. 1955. Tissue fractionation studies. 6. Intracellular distribution patterns of enzymes in rat-liver tissue. The Biochemical Journal 60, 604–617. 13249955 10.1042/bj0600604 PMC1216159

[eraf490-B23] Dethloff F, Orf I, Kopka J. 2017. Rapid in situ 13C tracing of sucrose utilization in Arabidopsis sink and source leaves. Plant Methods 13, 87. 29075313 10.1186/s13007-017-0239-6 PMC5648436

[eraf490-B24] Ding Z, Wang B, Moreno I, et al 2012. ER-localized auxin transporter PIN8 regulates auxin homeostasis and male gametophyte development in Arabidopsis. Nature Communications 3, 941. 10.1038/ncomms1941 22760640

[eraf490-B25] Dittmer TA, Stacey NJ, Sugimoto-Shirasu K, Richards EJ. 2007. *LITTLE NUCLEI* genes affecting nuclear morphology in *Arabidopsis thaliana*. The Plant Cell 19, 2793–2803. 17873096 10.1105/tpc.107.053231 PMC2048703

[eraf490-B26] Doležel J, Greilhuber J, Suda J. 2007. Estimation of nuclear DNA content in plants using flow cytometry. Nature Protocols 2, 2233–2244. 17853881 10.1038/nprot.2007.310

[eraf490-B27] Drakakaki G, van de Ven W, Pan S, et al 2012. Isolation and proteomic analysis of the SYP61 compartment reveal its role in exocytic trafficking in Arabidopsis. Cell Research 22, 413–424. 21826108 10.1038/cr.2011.129 PMC3271593

[eraf490-B28] Dubacq JP, Kader JC. 1978. Free flow electrophoresis of chloroplasts. Plant Physiology 61, 465–468. 16660315 10.1104/pp.61.3.465 PMC1091890

[eraf490-B29] Dvořáčková M, Fajkus J, Mozgová I, Pečinka A. 2024. Advances in plant chromatin. The Plant Journal 118, 1281–1283. 38814105 10.1111/tpj.16648

[eraf490-B30] Edwards GE, Robinson SP, Tyler NJ, Walker DA. 1978. Photosynthesis by isolated protoplasts, protoplast extracts, and chloroplasts of wheat: influence of orthophosphate, pyrophosphate, and adenylates. Plant Physiology 62, 313–319. 16660508 10.1104/pp.62.2.313 PMC1092112

[eraf490-B31] Eubel H, Lee CP, Kuo J, Meyer EH, Taylor NL, Millar AH. 2007. Free-flow electrophoresis for purification of plant mitochondria by surface charge. The Plant Journal 52, 583–594. 17727614 10.1111/j.1365-313X.2007.03253.x

[eraf490-B32] Eubel H, Meyer EH, Taylor NL, Bussell JD, O'Toole N, Heazlewood JL, Castleden I, Small ID, Smith SM, Millar AH. 2008. Novel proteins, putative membrane transporters, and an integrated metabolic network are revealed by quantitative proteomic analysis of Arabidopsis cell culture peroxisomes. Plant Physiology 148, 1809–1829. 18931141 10.1104/pp.108.129999 PMC2593673

[eraf490-B33] Farmer A, Thibivilliers S, Ryu KH, Schiefelbein J, Libault M. 2021. Single-nucleus RNA and ATAC sequencing reveals the impact of chromatin accessibility on gene expression in Arabidopsis roots at the single-cell level. Molecular Plant 14, 372–383. 33422696 10.1016/j.molp.2021.01.001

[eraf490-B34] Flores HE, Galston AW. 1984. Osmotic stress-induced polyamine accumulation in cereal leaves: I. Physiological parameters of the response. Plant Physiology 75, 102–109. 16663551 10.1104/pp.75.1.102 PMC1066843

[eraf490-B35] Fluckiger R, De Caroli M, Piro G, Dalessandro G, Neuhaus J-M, Di Sansebastiano G-P. 2003. Vacuolar system distribution in Arabidopsis tissues, visualized using GFP fusion proteins. Journal of Experimental Botany 54, 1577–1584. 12730271 10.1093/jxb/erg160

[eraf490-B36] Folta KM, Kaufman LS. 2006. Isolation of Arabidopsis nuclei and measurement of gene transcription rates using nuclear run-on assays. Nature Protocols 1, 3094–3100. 17406505 10.1038/nprot.2006.471

[eraf490-B37] Fürtauer L, Weckwerth W, Nägele T. 2016. A benchtop fractionation procedure for subcellular analysis of the plant metabolome. Frontiers in Plant Science 7, 1912. 28066469 10.3389/fpls.2016.01912 PMC5177628

[eraf490-B38] Gaiero P, Šimková H, Vrána J, Santiñaque FF, López-Carro B, Folle GA, van de Belt J, Peters SA, Doležel J, de Jong H. 2018. Intact DNA purified from flow-sorted nuclei unlocks the potential of next-generation genome mapping and assembly in Solanum species. MethodsX 5, 328–336. 30046519 10.1016/j.mex.2018.03.009 PMC6058011

[eraf490-B39] Galbraith DW, Harkins KR, Maddox JM, Ayres NM, Sharma DP, Firoozabady E. 1983. Rapid flow cytometric analysis of the cell cycle in intact plant tissues. Science 220, 1049–1051. 17754551 10.1126/science.220.4601.1049

[eraf490-B40] Garcia-Hernandez S, Morello-López J, Haslam R, et al 2025. Concerted transport and phosphorylation of diacylglycerol at ER–PM contact sites regulate phospholipid dynamics during stress. Proceedings of the National Academy of Sciences, USA 122, e2421334122. 10.1073/pnas.2421334122 PMC12167946 40455983

[eraf490-B41] Gilliard G, Huby E, Cordelier S, Ongena M, Dhondt-Cordelier S, Deleu M. 2021. Protoplast: a valuable toolbox to investigate plant stress perception and response. Frontiers in Plant Science 12, 749581. 34675954 10.3389/fpls.2021.749581 PMC8523952

[eraf490-B42] Godfrey WL, Rudd CJ, Iyer S, Recktenwald D. 2005. Purification of cellular and organelle populations by fluorescence-activated cell sorting for proteome analysis. In: Walker JM, ed. The proteomics protocols handbook. Springer protocols handbooks. Totowa NJ: Humana Press, 67–78.

[eraf490-B43] Grebe M, Xu J, Möbius W, Ueda T, Nakano A, Geuze HJ, Rook MB, Scheres B. 2003. Arabidopsis sterol endocytosis involves actin-mediated trafficking via ARA6-positive early endosomes. Current Biology 13, 1378–1387. 12932321 10.1016/s0960-9822(03)00538-4

[eraf490-B44] Gutzat R, Rembart K, Nussbaumer T, et al 2020. *Arabidopsis* shoot stem cells display dynamic transcription and DNA methylation patterns. The EMBO Journal 39, e103667. 32815560 10.15252/embj.2019103667 PMC7560203

[eraf490-B45] Haas C, Hegner R, Helbig K, Bartels K, Bley T, Weber J. 2016. Two parametric cell cycle analyses of plant cell suspension cultures with fragile, isolated nuclei to investigate heterogeneity in growth of batch cultivations. Biotechnology and Bioengineering 113, 1244–1250. 26614913 10.1002/bit.25894

[eraf490-B46] Hamamoto S, Uozumi N. 2014. Organelle-localized potassium transport systems in plants. Journal of Plant Physiology 171, 743–747. 24810770 10.1016/j.jplph.2013.09.022

[eraf490-B47] Harkins KR, Galbraith DW. 1987. Factors governing the flow cytometric analysis and sorting of large biological particles. Cytometry 8, 60–70. 3803096 10.1002/cyto.990080110

[eraf490-B48] Hayashi K, Nakamura S, Fukunaga S, Nishimura T, Jenness MK, Murphy AS, Motose H, Nozaki H, Furutani M, Aoyama T. 2014. Auxin transport sites are visualized in planta using fluorescent auxin analogs. Proceedings of the National Academy of Sciences, USA 111, 11557–11562. 10.1073/pnas.1408960111 PMC4128153 25049419

[eraf490-B49] Hu J, Baker A, Bartel B, Linka N, Mullen RT, Reumann S, Zolman BK. 2012. Plant peroxisomes: biogenesis and function. The Plant Cell 24, 2279–2303. 22669882 10.1105/tpc.112.096586 PMC3406917

[eraf490-B50] Huang AHC, Beevers H. 1971. Isolation of microbodies from plant tissues. Plant Physiology 48, 637–641. 16657851 10.1104/pp.48.5.637 PMC396919

[eraf490-B51] Huber LA, Pfaller K, Vietor I. 2003. Organelle proteomics: implications for subcellular fractionation in proteomics. Circulation Research 92, 962–968. 12750306 10.1161/01.RES.0000071748.48338.25

[eraf490-B52] International Wheat Genome Sequencing Consortium . 2018. Shifting the limits in wheat research and breeding using a fully annotated reference genome. Science 361, eaar7191. 30115783 10.1126/science.aar7191

[eraf490-B53] Islinger M, Wildgruber R, Völkl A. 2018. Preparative free-flow electrophoresis, a versatile technology complementing gradient centrifugation in the isolation of highly purified cell organelles. Electrophoresis 39, 2288–2299. 29761848 10.1002/elps.201800187

[eraf490-B54] Keech O, Dizengremel P, Gardeström P. 2005. Preparation of leaf mitochondria from *Arabidopsis thaliana*. Physiologia Plantarum 124, 403–409.

[eraf490-B55] Kriechbaumer V . 2024. Preparation of ER microsomes from *Arabidopsis thaliana*. Methods in Molecular Biology 2772, 129–135. 38411810 10.1007/978-1-0716-3710-4_9

[eraf490-B56] Kuhnert F, Stefanski A, Overbeck N, Drews L, Reichert AS, Stühler K, Weber APM. 2020. Rapid single-step affinity purification of HA-tagged plant mitochondria. Plant Physiology 182, 692–706. 31818904 10.1104/pp.19.00732 PMC6997695

[eraf490-B57] Kunz HH, Gierth M, Herdean A, Satoh-Cruz M, Kramer DM, Spetea C, Schroeder JI. 2014. Plastidial transporters KEA1, -2, and -3 are essential for chloroplast osmoregulation, integrity, and pH regulation in *Arabidopsis*. Proceedings of the National Academy of Sciences, USA 111, 7480–7485. 10.1073/pnas.1323899111 PMC4034250 24794527

[eraf490-B58] Lee YH, Tan HT, Chung MCM. 2010. Subcellular fractionation methods and strategies for proteomics. Proteomics 10, 3935–3956. 21080488 10.1002/pmic.201000289

[eraf490-B59] Leigh RA, Branton D. 1976. Isolation of vacuoles from root storage tissue of *Beta vulgaris* L. Plant Physiology 58, 656–662. 16659738 10.1104/pp.58.5.656 PMC542277

[eraf490-B60] Liang Z, Yu C, Huang AH. 1982. Isolation of spinach leaf peroxisomes in 0.25 molar sucrose solution by percoll density gradient centrifugation. Plant Physiology 70, 1210–1212. 16662639 10.1104/pp.70.4.1210 PMC1065851

[eraf490-B61] Logan DC, Leaver CJ. 2000. Mitochondria-targeted GFP highlights the heterogeneity of mitochondrial shape, size and movement within living plant cells. Journal of Experimental Botany 51, 865–871. 10948212

[eraf490-B62] Loureiro J, Kron P, Temsch EM, Koutecký P, Lopes S, Castro M, Castro S. 2021. Isolation of plant nuclei for estimation of nuclear DNA content: overview and best practices. Cytometry Part A 99, 318–327. 10.1002/cyto.a.24331 33751820

[eraf490-B63] Ma S, Quist TM, Ulanov A, Joly R, Bohnert HJ. 2004. Loss of TIP1;1 aquaporin in Arabidopsis leads to cell and plant death. The Plant Journal 40, 845–859. 15584951 10.1111/j.1365-313X.2004.02265.x

[eraf490-B64] Markham JE, Molino D, Gissot L, Bellec Y, Hématy K, Marion J, Belcram K, Palauqui JC, Satiat-Jeunemaître B, Faure JD. 2011. Sphingolipids containing very-long-chain fatty acids define a secretory pathway for specific polar plasma membrane protein targeting in *Arabidopsis*. The Plant Cell 23, 2362–2378. 21666002 10.1105/tpc.110.080473 PMC3160045

[eraf490-B65] Matsushima R, Hayashi Y, Kondo M, Shimada T, Nishimura M, Hara-Nishimura I. 2002. An endoplasmic reticulum-derived structure that is induced under stress conditions in Arabidopsis. Plant Physiology 130, 1807–1814. 12481064 10.1104/pp.009464 PMC166692

[eraf490-B66] McKeown P, Pendle AF, Shaw PJ. 2008. Preparation of arabidopsis nuclei and nucleoli. Methods in Molecular Biology 463, 67–75. 18951161 10.1007/978-1-59745-406-3_5

[eraf490-B67] Miao Y, Li KY, Li HY, Yao X, Jiang L. 2008. The vacuolar transport of aleurain–GFP and 2S albumin–GFP fusions is mediated by the same pre-vacuolar compartments in tobacco BY-2 and Arabidopsis suspension cultured cells. The Plant Journal 56, 824–839. 18680561 10.1111/j.1365-313X.2008.03645.x

[eraf490-B68] Miflin BJ, Beevers H. 1974. Isolation of intact plastids from a range of plant tissues. Plant Physiology 53, 870–874. 16658807 10.1104/pp.53.6.870 PMC541465

[eraf490-B69] Miller LA, Romani RJ. 1966. Sucrose density gradient distribution of mitochondrial protein and enzymes from preclimacteric and climacteric pears. Plant Physiology 41, 411–414. 16656269 10.1104/pp.41.3.411 PMC1086357

[eraf490-B70] Moreno-Romero J, Santos-González J, Hennig L, Köhler C. 2017. Applying the INTACT method to purify endosperm nuclei and to generate parental-specific epigenome profiles. Nature Protocols 12, 238–254. 28055034 10.1038/nprot.2016.167

[eraf490-B71] Muñoz P, Norambuena L, Orellana A. 1996. Evidence for a UDP-glucose transporter in Golgi apparatus-derived vesicles from pea and its possible role in polysaccharide biosynthesis. Plant Physiology 112, 1585–1594. 12226465 10.1104/pp.112.4.1585 PMC158091

[eraf490-B72] Nelson BK, Cai X, Nebenführ A. 2007. A multicolored set of *in vivo* organelle markers for co-localization studies in Arabidopsis and other plants. The Plant Journal 51, 1126–1136. 17666025 10.1111/j.1365-313X.2007.03212.x

[eraf490-B73] Niehaus M, Straube H, Künzler P, Rugen N, Hegermann J, Giavalisco P, Eubel H, Witte CP, Herde M. 2020. Rapid affinity purification of tagged plant mitochondria (mito-AP) for metabolome and proteome analyses. Plant Physiology 182, 1194–1210. 31911558 10.1104/pp.19.00736 PMC7054873

[eraf490-B74] Nitta N, Sugimura T, Isozaki A, et al 2018. Intelligent image-activated cell sorting. Cell 175, 266–276.e13. 30166209 10.1016/j.cell.2018.08.028

[eraf490-B75] Oda Y, Fukuda H. 2011. Dynamics of Arabidopsis SUN proteins during mitosis and their involvement in nuclear shaping. The Plant Journal 66, 629–641. 21294795 10.1111/j.1365-313X.2011.04523.x

[eraf490-B76] Okekeogbu IO, Aryal UK, Fernández-Niño SMG, Penning BW, Heazlewood JL, McCann MC, Carpita NC. 2019. Differential distributions of trafficking and signaling proteins of the maize ER–Golgi apparatus. Plant Signaling and Behavior 14, 1672513. 31564200 10.1080/15592324.2019.1672513 PMC6866702

[eraf490-B77] Parsons HT, Christiansen K, Knierim B, et al 2012. Isolation and proteomic characterization of the Arabidopsis Golgi defines functional and novel components involved in plant cell wall biosynthesis. Plant Physiology 159, 12–26. 22430844 10.1104/pp.111.193151 PMC3375956

[eraf490-B78] Petersson SV, Johansson AI, Kowalczyk M, Makoveychuk A, Wang JY, Moritz T, Grebe M, Benfey PN, Sandberg G, Ljung K. 2009. An auxin gradient and maximum in the *Arabidopsis* root apex shown by high-resolution cell-specific analysis of IAA distribution and synthesis. The Plant Cell 21, 1659–1668. 19491238 10.1105/tpc.109.066480 PMC2714926

[eraf490-B79] Petrovská B, Jeřábková H, Chamrád I, Vrána J, Lenobel R, Uřinovská J, Sebela M, Doležel J. 2014. Proteomic analysis of barley cell nuclei purified by flow sorting. Cytogenetic and Genome Research 143, 78–86. 25059295 10.1159/000365311

[eraf490-B80] Pfündel E, Meister A. 1996. Flow cytometry of mesophyll and bundle sheath chloroplast thylakoids of maize (*Zea mays* L.). Cytometry 23, 97–105. 8742167 10.1002/(SICI)1097-0320(19960201)23:2<97::AID-CYTO2>3.0.CO;2-I

[eraf490-B81] Piechulla B, Heldt H-W. 2025. Plant biochemistry. Cambridge: Academic Press.

[eraf490-B82] Reumann S, Babujee L, Ma C, Wienkoop S, Siemsen T, Antonicelli GE, Rasche N, Lüder F, Weckwerth W, Jahn O. 2007. Proteome analysis of *Arabidopsis* leaf peroxisomes reveals novel targeting peptides, metabolic pathways, and defense mechanisms. The Plant Cell 19, 3170–3193. 17951448 10.1105/tpc.107.050989 PMC2174697

[eraf490-B83] Reumann S, Lisik P. 2017. Isolation of Arabidopsis leaf peroxisomes and the peroxisomal membrane. Methods in Molecular Biology 1511, 97–112. 27730605 10.1007/978-1-4939-6533-5_8

[eraf490-B84] Rho JH, Chipchase MI. 1962. Incorporation of tritiated cytidine into ribonucleic acid by isolated pea nuclei. Journal of Cell Biology 14, 183–192. 14491776 10.1083/jcb.14.2.183 PMC2106106

[eraf490-B85] Robert S, Zouhar J, Carter CJ, Raikhel N. 2007. Isolation of intact vacuoles from Arabidopsis rosette leaf-derived protoplasts. Nature Protocols 2, 259–262. 17406583 10.1038/nprot.2007.26

[eraf490-B86] Rodrigues JMP, Pereira CS, Fontes N, Gerós H, Côrte-Real M. 2018. Flow cytometry and fluorescence microscopy as tools for structural and functional analysis of vacuoles isolated from yeast and plant cells. Methods in Molecular Biology 1789, 101–115. 29916074 10.1007/978-1-4939-7856-4_8

[eraf490-B87] Rodrigues JMP, Silva RD, Noronha H, Pedras A, Gerós H, Côrte-Real M. 2013. Flow cytometry as a novel tool for structural and functional characterization of isolated yeast vacuoles. Microbiology 159, 848–856. 23449920 10.1099/mic.0.062570-0

[eraf490-B88] Rodríguez-Rosales MP, Gálvez FJ, Huertas R, Aranda MN, Baghour M, Cagnac O, Venema K. 2009. Plant NHX cation/proton antiporters. Plant Signaling & Behavior 4, 265–276. 19794841 10.4161/psb.4.4.7919 PMC2664485

[eraf490-B89] Rogers ED, Jackson T, Moussaieff A, Aharoni A, Benfey PN. 2012. Cell type-specific transcriptional profiling: implications for metabolite profiling. The Plant Journal 70, 5–17. 22449039 10.1111/j.1365-313X.2012.04888.x PMC3315153

[eraf490-B90] Šafář J, Noa-Carrazana JC, Vrána J, et al 2004. Creation of a BAC resource to study the structure and evolution of the banana (*Musa balbisiana*) genome. Genome 47, 1182–1191. 15644977 10.1139/g04-062

[eraf490-B91] Saunders JA . 1979. Investigations of vacuoles isolated from tobacco: I. Quantitation of nicotine. Plant Physiology 64, 74–78. 16660918 10.1104/pp.64.1.74 PMC543027

[eraf490-B92] Saxena PK, Fowke LC, King J. 1985. An efficient procedure for isolation of nuclei from plant protoplasts. Protoplasma 128, 184–189.

[eraf490-B93] Schraivogel D, Kuhn TM, Rauscher B, et al 2022. High-speed fluorescence image-enabled cell sorting. Science 375, 315–320. 35050652 10.1126/science.abj3013 PMC7613231

[eraf490-B94] Schulz A, Knoetzel J, Scheller HV, Mant A. 2004. Uptake of a fluorescent dye as a swift and simple indicator of organelle intactness: import-competent chloroplasts from soil-grown *Arabidopsis*. Journal of Histochemistry and Cytochemistry 52, 701–704. 15100247 10.1177/002215540405200514

[eraf490-B95] Seigneurin-Berny D, Salvi D, Dorne AJ, Joyard J, Rolland N. 2008. Percoll-purified and photosynthetically active chloroplasts from *Arabidopsis thaliana* leaves. Plant Physiology and Biochemistry 46, 951–955. 18707896 10.1016/j.plaphy.2008.06.009

[eraf490-B96] Shapiro HM . 2024. Flow cytometry: the glass is half full. Methods in Molecular Biology 2779, 1–10. 38526779 10.1007/978-1-0716-3738-8_1

[eraf490-B97] Sharma M, Bennewitz B, Klösgen RB. 2018. Dual or not dual? Comparative analysis of fluorescence microscopy-based approaches to study organelle targeting specificity of nuclear-encoded plant proteins. Frontiers in Plant Science 9, 1350. 30298079 10.3389/fpls.2018.01350 PMC6160753

[eraf490-B98] Shav-Tal Y, Blechman J, Darzacq X, Montagna C, Dye BT, Patton JG, Singer RH, Zipori D. 2005. Dynamic sorting of nuclear components into distinct nucleolar caps during transcriptional inhibition. Molecular Biology of the Cell 16, 2395–2413. 15758027 10.1091/mbc.E04-11-0992 PMC1087244

[eraf490-B99] Šimková H, Číhalíková J, Vrána J, Lysák MA, Dolezel J. 2003. Preparation of HMW DNA from plant nuclei and chromosomes isolated from root tips. Biologia Plantarum 46, 369–373.

[eraf490-B100] Skalický V, Antoniadi I, Pěnčík A, et al 2023. Fluorescence-activated multi-organelle mapping of subcellular plant hormone distribution. The Plant Journal 116, 1825–1841. 37682018 10.1111/tpj.16456

[eraf490-B101] Skalický V, Kubeš M, Napier R, Novák O. 2018. Auxins and cytokinins—the role of subcellular organization on homeostasis. International Journal of Molecular Sciences 19, 3115. 30314316 10.3390/ijms19103115 PMC6213326

[eraf490-B102] Skalický V, Vojtková T, Pěnčík A, Vrána J, Juzoń K, Koláčková V, Sedlářová M, Kubeš MF, Novák O. 2021. Auxin metabolite profiling in isolated and intact plant nuclei. International Journal of Molecular Sciences 22, 12369. 34830250 10.3390/ijms222212369 PMC8620152

[eraf490-B103] Soll J, Schultz G, Joyard J, Douce R, Block MA. 1985. Localization and synthesis of prenylquinones in isolated outer and inner envelope membranes from spinach chloroplasts. Archives of Biochemistry and Biophysics 238, 290–299. 3985624 10.1016/0003-9861(85)90167-5

[eraf490-B104] Somerville CR, Somerville SC, Ogren WL. 1981. Isolation of photosynthetically active protoplasts and chloroplasts from *Arabidopsis thaliana*. Plant Science Letters 21, 89–96.

[eraf490-B105] Song Y, Hao Y, Sun A, et al 2006. Sample preparation project for the subcellular proteome of mouse liver. Proteomics 6, 5269–5277. 16941572 10.1002/pmic.200500893

[eraf490-B106] Steen HB . 2000. Flow cytometry of bacteria: glimpses from the past with a view to the future. Journal of Microbiological Methods 42, 65–74. 11000432 10.1016/s0167-7012(00)00177-9

[eraf490-B107] Stitt M, Lilley RM, Gerhardt R, Heldt HW. 1989. Metabolite levels in specific cells and subcellular compartments of plant-leaves. Methods in Enzymology 174, 518–552.

[eraf490-B108] Thibivilliers S, Anderson D, Libault M. 2020. Isolation of plant root nuclei for single cell RNA sequencing. Current Protocols in Plant Biology 5, e20120. 33034428 10.1002/cppb.20120

[eraf490-B109] van Wijk KJ, Baginsky S. 2011. Plastid proteomics in higher plants: current state and future goals. Plant Physiology 155, 1578–1588. 21350036 10.1104/pp.111.172932 PMC3091083

[eraf490-B110] Včelařová L, Skalický V, Chamrád I, Lenobel R, Kubeš MF, Pěnčík A, Novák O. 2021. Auxin metabolome profiling in the Arabidopsis endoplasmic reticulum using an optimised organelle isolation protocol. International Journal of Molecular Sciences 22, 9370. 34502279 10.3390/ijms22179370 PMC8431077

[eraf490-B111] Viotti C, Krüger F, Krebs M, et al 2013. The endoplasmic reticulum is the main membrane source for biogenesis of the lytic vacuole in *Arabidopsis*. The Plant Cell 25, 3434–3449. 24014545 10.1105/tpc.113.114827 PMC3809542

[eraf490-B112] Wada M, Suetsugu N. 2004. Plant organelle positioning. Current Opinion in Plant Biology 7, 626–631. 15491910 10.1016/j.pbi.2004.09.005

[eraf490-B113] Walker DA . 1964. Improved rates of carbon dioxide fixation by illuminated chloroplasts. The Biochemical Journal 92, 22C–23C. 10.1042/bj0920022c 5837433

[eraf490-B114] Wang H, Han S, Siao W, et al 2015. Arabidopsis synaptotagmin 2 participates in pollen germination and tube growth and is delivered to plasma membrane via conventional secretion. Molecular Plant 8, 1737–1750. 26384245 10.1016/j.molp.2015.09.003

[eraf490-B115] Wilkop T, Pattathil S, Ren G, Davis DJ, Bao W, Duan D, Peralta AG, Domozych DS, Hahn MG, Drakakaki G. 2019. A hybrid approach enabling large-scale glycomic analysis of post-Golgi vesicles reveals a transport route for polysaccharides. The Plant Cell 31, 627–644. 30760563 10.1105/tpc.18.00854 PMC6482635

[eraf490-B116] Wolf PG, Karol KG, Mandoli DF, Kuehl J, Arumuganathan K, Ellis MW, Mishler BD, Kelch DG, Olmstead RG, Boore JL. 2005. The first complete chloroplast genome sequence of a lycophyte, *Huperzia lucidula* (Lycopodiaceae). Gene 350, 117–128. 15788152 10.1016/j.gene.2005.01.018

[eraf490-B117] Woodson JD, Chory J. 2008. Coordination of gene expression between organellar and nuclear genomes. Nature Reviews. Genetics 9, 383–395. 10.1038/nrg2348 PMC4854206 18368053

[eraf490-B118] Wulfetange K, Lomin SN, Romanov GA, Stolz A, Heyl A, Schmülling T. 2011. The cytokinin receptors of Arabidopsis are located mainly to the endoplasmic reticulum. Plant Physiology 156, 1808–1818. 21709172 10.1104/pp.111.180539 PMC3149959

[eraf490-B119] Yao N, Eisfelder BJ, Marvin J, Greenberg JT. 2004. The mitochondrion—an organelle commonly involved in programmed cell death in *Arabidopsis thaliana*. The Plant Journal 40, 596–610. 15500474 10.1111/j.1365-313X.2004.02239.x

[eraf490-B120] Zalabák D, Johnová P, Plíhal O, Šenková K, Šamajová O, Jiskrová E, Novák O, Jackson D, Mohanty A, Galuszka P. 2016. Maize cytokinin dehydrogenase isozymes are localized predominantly to the vacuoles. Plant Physiology and Biochemisty 104, 114–124. 10.1016/j.plaphy.2016.03.013 27031423

